# Adherence to Long-Acting Inhaler Use for Initial Treatment and Acute Exacerbation of Chronic Obstructive Pulmonary Disease: A Retrospective Cohort Study

**DOI:** 10.3390/jpm12122073

**Published:** 2022-12-15

**Authors:** Hee-Sook Suh, Min-Seok Chang, Iseul Yu, Sunmin Park, Ji-Ho Lee, Seok Jeong Lee, Won-Yeon Lee, Suk Joong Yong, Sang-Ha Kim

**Affiliations:** 1 Policy Research Department, Health Insurance Review and Assessment Service, 60 Hyeoksinro, Wonju 26465, Gangwon, Republic of Korea; 2 Department of Internal Medicine, Yonsei University Wonju College of Medicine, 20 Ilsanro, Wonju 26426, Gangwon, Republic of Korea

**Keywords:** chronic obstructive pulmonary disease, medication adherence, acute exacerbation, long-acting inhaler

## Abstract

We aimed to determine the effect of long-acting inhaler use adherence on acute exacerbations in treatment-naïve patients with chronic obstructive pulmonary disease (COPD) using claims data from the Korean Health Insurance Review and Assessment Service from July 2015–December 2016. Patients with COPD aged ≥ 40 years who used long-acting inhalers were enrolled and observed for 6 months. Medication adherence was determined by the medication possession ratio (MPR); patients were categorized to adherence (MPR ≥ 80%) and non-adherence (MPR < 80%) groups. Ultimately, 3959 patients were enrolled: 60.4% and 39.6% in the adherence and non-adherence groups, respectively. The relative risk of acute exacerbation in the non-adherence group was 1.58 (95% confidence interval [CI] 1.25–1.99) compared with the adherence group. The adjusted logistic regression analysis revealed a relative risk of acute exacerbation in the non-adherence vs. adherence group of 1.68 (95% CI 1.32–2.14) regarding the number of inhalers used. Poor adherence to long-acting inhalers influenced increased acute exacerbation rates among patients with COPD. The acute exacerbation of COPD risk requiring hospitalization or ED visits was high in the non-adherence group, suggesting that efforts to improve medication adherence may help reduce COPD exacerbations even in the initial management of treatment-naïve patients.

## 1. Introduction

Chronic obstructive pulmonary disease (COPD) is characterized by airflow blockage leading to breathing difficulties and is a cause of serious social and economic burdens globally. Although exacerbations during COPD treatment are associated with increased rates of hospitalization, readmission, and disease progression [1], medication adherence is poor compared with that observed in other chronic diseases, such as diabetes, hypertension, hypercholesterolemia, osteoporosis, and depression [2]. In clinical trials, the adherence rate for inhaled medication approached 80% [3]; however, in real-world clinical situations the adherence rate ranged from 23% to 60% [4,5].

For patients at high risk of exacerbations, inhaled long-acting bronchodilators that can be supplemented with inhaled corticosteroids are recommended for the long-term treatment plan [6]. Patients’ adherence to prescribed long-acting inhalers for maintenance can influence COPD symptoms, physical activities, acute exacerbations, and disease progression. Patients with COPD are vulnerable to medication adherence due to disease chronicity, multidrug use, and time to symptom relief [7].

A few studies have examined the effect of medication adherence on COPD exacerbations in real-world situations [5,8,9,10,11], although studies on the relationship between medication (long-acting inhaler) adherence and hospitalizations or emergency department (ED) visits caused by acute exacerbation, especially in newly diagnosed patients, are lacking. Using Korean national health insurance claims data, this study aimed to investigate the impact of medication adherence on exacerbations in patients with COPD.

## 2. Materials and Methods

### 2.1. Dataset and Study Population

We conducted this retrospective observational cohort study from 1 July 2015, to 31 December 2016, using the Korean Health Insurance Review and Assessment Service (HIRA) database, which contains healthcare service information of 50 million beneficiaries. The dataset contained information on drugs, tests, surgeries, and procedures submitted by the hospital to HIRA for medical expense claims. Further, the HIRA database contains information on patients’ sex, age, diagnoses, and specialty and department of healthcare provider, as well as an alternative key to identify individuals for privacy, and de-identification of individual patient data. The HIRA Institutional Review Board approved this study and waived the requirement of informed patient consent due to the retrospective design of the study (2018-019-002).

**Figure 1:**
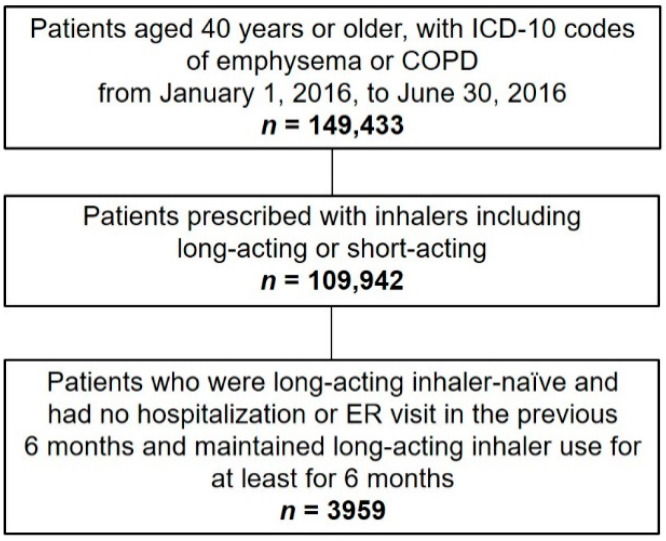
Flow chart describing the details for the number of patients in this study. ICD-10, International Classification of Diseases-10th Revision; COPD, chronic obstructive pulmonary disease; ER, emergency room.

An operational definition for extracting patients with COPD from the HIRA datasets was used [12,13]. We searched for patients with COPD as a primary or secondary diagnosis based on the International Classification of Diseases-10th Revision (ICD-10) codes and prescribed medication. All patients aged 40 years or older, with ICD-10 codes of emphysema or COPD (J43.x, J44.x, except J43.0) and at least two claims for COPD medications, including long-acting muscarinic antagonist, long-acting beta-2 agonist (LABA), inhaled corticosteroid and LABA, or short-acting bronchodilators were included. The included patients were first prescribed the long-acting inhalers (between 1 January 2016, and 30 June 2016) as maintenance medication (maintained over a period of at least 6 months). These long-acting inhaler-naïve patients had no hospitalization and ED visits (COPD-related) for 6 months before the first prescription. Long-acting inhalers for maintenance therapy included either a single agent or a combination of agents (long-acting muscarinic antagonist, LABA, or inhaled corticosteroid) (Figure 1).

### 2.2. Medication Adherence and Comorbidities

Medication adherence to long-acting inhalers was calculated using the medication possession ratio (MPR). The MPR is defined as the proportion of days covered by long-acting inhalers during the study period. For the MPR calculation, the treatment period was the first outpatient visit 6 months from the initial prescription date, while the number of days covered by the inhaled medication was calculated by the number of inhalers prescribed multiplied by the days of inhaler dosing for the pack unit. MPRs of ≥80% and <80% were defined as good and poor medication adherence, respectively [14,15,16].

The common and clinically significant COPD-associated comorbidities analyzed (from the primary and entire secondary diagnoses per claim data) included ischemic heart disease (I20.0–I25), heart failure (I50), osteoporosis (M80–M82), and depression (F32 and F33). The number of comorbidities was classified into five categories (0 to 4).

### 2.3. Outcome Variables

We evaluated the effect of medication (long-acting inhalers) adherence on the development of COPD exacerbation during the 6-month follow-up period after the first prescription date. COPD exacerbation was defined as hospitalizations or ED visits with a primary or secondary diagnosis of emphysema or COPD (J43.x, J44.x, except J43.0 from ICD-10 codes).

### 2.4. Statistical Analyses

The chi-square test and independent t-test were used to determine the differences in categorical variables and continuous data between the patient groups (with and without COPD exacerbation) according to medication adherence. We conducted univariable and multivariable analyses to identify the significant factors for COPD exacerbation. The log-rank test was used to compare the occurrence of acute exacerbation in patients with COPD with and without risk factors during the study period (6 months), and the outcomes were plotted as Kaplan-Meier curves. Statistical analyses were conducted using SAS 9.3 (SAS Institute, Inc., Cary, NC, USA).

## 3. Results

### 3.1. Study Population Baseline Characteristics according to Medication Adherence

The datasets of a total of 3959 patients (23.4% women, 76.6% men, >85% aged ≥60 years) were analyzed (Figure 1, Table 1): 60.4% of patients showed good medication adherence (MPR ≥ 80%). The percentage of patients with no, one, two, three, or four comorbid disease(s) was 54.8%, 30.2%, 12.0%, 2.6%, and 0.3%, respectively. Most patients (81.8%) used one inhaler, and the total number of patients who displayed acute exacerbation during the 6-month follow-up period was 312 (7.9%).

The proportion of male patients was significantly higher (78.3%) in the adherence group (MPR ≥ 80%) than in the non-adherence (MPR < 80%) group (74.1%). The adherence group had more patients aged 40–60 years (44.8% vs. 37.8%) and fewer patients aged ≥ 70 years (55.3% vs. 62.2%) than the non-adherence group. Further, the proportion of patients who used one inhaler was higher (84.8%) in the non-adherence group than in the adherence group (79.9%), while the number of patients who used ≥ 2 inhalers was significantly higher (20.1%) in the adherence group than in the non-adherence group (15.2%, *p* < 0.0001). The acute exacerbation incidences were significantly higher (9.9%) in the non-adherence group than in the adherence group (6.5%, *p* < 0.0001). No differences were observed between the adherence and non-adherence groups in terms of the healthcare coverage type, use of outpatient clinic, and number of comorbid diseases.

### 3.2. Differences in Clinical Characteristics and Adherence to Long-Acting Inhalers according to COPD Exacerbation Incidence

Among the patients with COPD divided into the incident and non-incident groups of acute exacerbation, the proportion of men was significantly higher (82.7%) in the incident group than in the non-incident group (76.1%) (Table 2). The proportion of patients who visited ≥ 2 and one outpatient clinic(s) was higher in the incident and non-incident group, respectively.

Further, patients with ≥2 comorbid diseases and who used ≥2 inhalers showed higher percentages in the incident group than in the non-incident group; no significant difference was observed in terms of the healthcare coverage type.

The proportions of patients with poor (MPR < 80%) and good (MPR ≥ 80%) medication adherence were higher in the incident and non-incident group, respectively (Table 2).

### 3.3. Impact of Medication Adherence to Long-Acting Inhalers on COPD Exacerbation

The analysis of the risk factors for acute exacerbation in patients with COPD showed that the relative risk of acute exacerbation in the non-adherence group (MPR < 80%) was 1.58 (95% confidence interval [CI] 1.25–1.99) in comparison with the adherence group (MPR ≥ 80%). Relative risks of acute exacerbation in male vs. female patients, patients aged 70–79 years, and ≥80 years (vs. aged 40–49 years) were 1.50 (95% CI 1.11–2.03), 10.89 (95% CI 1.51–78.61), and 14.39 (95% CI 1.98–104.51), respectively, and 1.35 (95% CI 1.03–1.78), 2.26 (95% CI 1.64–3.13), 4.71 (95% CI 2.89–7.68), and 5.23 (95% CI 1.40–19.56) in patients with 1, 2, 3, and 4 comorbid disease(s), respectively. These results suggested that a greater number of comorbid diseases is associated with increased risk of exacerbation. In addition, the relative risk of acute exacerbation in patients who used ≥2 inhalers (vs. those who used a single inhaler) was 2.50 (95% CI 1.95–3.21) (Table 3).

**Figure 2:**
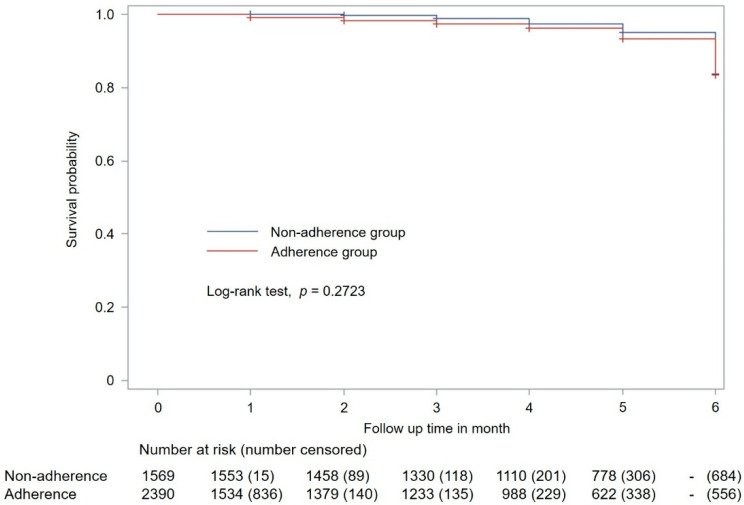
Kaplan–Meier plot showing acute exacerbation occurrence in patients with COPD. The adherence and non-adherence groups are classified based on medication adherence to long-acting inhalers. COPD, chronic obstructive pulmonary disease.

When logistic regression analysis was conducted after adjusting for the patient-related variables, the relative risk of acute exacerbation in the non-adherence group (vs. the adherence group) was 1.55 (95% CI 1.22–1.95), 1.57 (95% CI 1.24–1.99), and 1.68 (95% CI 1.32–2.14) in models 1 (sex- and age-adjusted), 2 (model 1 variables and comorbid diseases), and 3 (model 2 variables and number of inhalers used), respectively. These results suggested that the risk of acute exacerbation in patients with COPD was low and statistically significant in the adherence group (Table 4). Further, the Kaplan–Meier survival curves on the occurrence of acute exacerbation showed no significant difference by the log-rank test between the adherence and non-adherence groups (*p* = 0.2723) (Figure 2). When the adherence and non-adherence groups were followed for 6 months, no difference in the occurrence of acute exacerbation over time was observed between the two groups.

## 4. Discussion

This study showed that the risk of hospitalization or ED visits was higher for patients (treatment-naïve) with COPD with low medication (long-acting inhalers) adherence in a real-world scenario. The proportion of patients showing good compliance (60.4%) was consistent with that of previous reports of 10.8% [17], 39.0% [11], and 64.9% [15]. However, among these studies, only one [15] reported a high proportion of good compliance in treatment-naïve patients with COPD. Our study also included patients who had their first COPD drug claim during the first 6-month period following the diagnosis. Although drug adherence in real-world clinical practice has been reported in various clinical settings, adherence in these settings is expected to be relatively high for first prescriptions or short follow-up periods.

This study determined that 7.9% of the patients who had no previous hospitalization or ED visits due to COPD were hospitalized or visited the ED during the 6-month follow-up. In a previous large observational cohort study, the proportion of patients who were hospitalized during the first year of follow-up was 7, 18, and 33% in the Global Initiative for Chronic Obstructive Lung Disease (GOLD) stages 2, 3, and 4, respectively [18]. The authors confirmed that one of the best predictors of exacerbations in their study was exacerbation history. In another similar study that included all the GOLD stages (1 to 4), 13% had at least one and 9% of patients had two or more exacerbations in the first year of follow-up [19]: the authors defined the exacerbations as either moderate (treated with steroids or antibiotics) or severe (required hospital admission). We used this classification in our study to identify severe acute exacerbations that occurred during a relatively short follow-up period of 6 months.

Previous studies have reported that factors such as female sex, high comorbidities, better lung function (higher forced expiratory volume in 1 s), long duration of inhaler use, and high frequency of daily inhaler use were associated with poor adherence [5,10,15,17,20,21,22]. Consistently, we also observed that low adherence was associated with female sex, >70 years of age, and lower number of inhalers in use. In our study, patients who used only one inhaler for maintenance tended to have milder symptoms compared to patients who used two or more inhalers.

COPD exacerbations are shown to be associated with increased medical expenses (because of hospitalizations or ED visits) and mortality [18]. Several studies have demonstrated increased hospitalizations, mortality, healthcare resource utilization, and costs for non-adherent patients as compared with adherent patients [3,11,14]. A post hoc analysis of the “TOwards a Revolution in COPD Health” (TORCH) study revealed that patients with COPD and higher adherence to maintenance medications experienced lower annual hospitalization (0.15 vs. 0.27) and mortality (11.3% vs. 26.4%) rates compared to patients with lower adherence [3]. A retrospective cross-sectional study using claims data confirmed that good adherence was associated with lower hospital admission rates for exacerbations and Medicare costs including inpatient and outpatient costs [11]. Our study also confirmed that patients with low adherence had increased incidences of acute exacerbations.

Comorbidities constitute another important factor for the economic burden of COPD [23]. Among patients with COPD, the number of patients with one or more comorbidities varied from 26.1% to 73.2% [23,24]. Common comorbidities include cardiovascular diseases such as ischemic heart disease, heart failure, metabolic syndrome, osteoporosis, musculoskeletal disorders, anxiety, depression, and lung cancer [25]. In our study, four comorbidities (ischemic heart disease, heart failure, osteoporosis, and depression) were identified, and 45.2% of the patients showed one or more of these comorbidities. Consistent with previous reports, a higher number of comorbidities was correlated with frequent COPD exacerbations [20,23,24]. Effective management of comorbidities from the beginning of COPD treatment would reduce medical costs associated with exacerbations.

In this study, in addition to low medication adherence, it was confirmed that male sex, >70 years of age, number of comorbidities, and use of two or more inhalers were risk factors associated with acute exacerbations. Among these risk factors, the number of inhalers used and adherence to inhalers are factors that can be sufficiently modified during the course of treatment. A systematic literature review analyzed the difference between single and multiple inhalers for COPD treatment [21]. An analysis of economic impacts from retrospective and prospective studies revealed that the use of single-inhaler therapy was more cost-effective and reduced the use of healthcare resources, in comparison with multiple-inhaler therapy [21]. In addition, the use of the correct inhalation technique also affects the occurrence of acute exacerbations [26]. However, our study could not confirm this because it utilized nationwide claims data. A recent randomized controlled trial showed significantly improved adherence with the use of an audio-reminder generated by an application or alarm clock [27]. Hence, the use of a single inhaler and improving adherence to inhalers from the beginning of treatment may reduce the frequency of exacerbations in patients with COPD.

This study has some limitations. First, disease severity was not analyzed because the study was conducted using healthcare claims data without assessment of direct clinical data such as spirometry results and smoking habits. Second, since medication adherence was measured by MPR, it was not possible to determine whether the patient had inhaled all the medication. The possibility cannot be excluded that the number of days of medication administration may have been overestimated. Additionally, even if the patients were prescribed and had inhalers, we could not confirm whether the inhalers were being used correctly. Finally, the follow-up period of 6 months was not sufficient, particularly because long-term use of inhalers in patients with COPD is one of the factors influencing medication adherence and exacerbation occurrence; hence, future studies are required with longer follow-up times.

## 5. Conclusions

This study revealed that poor medication adherence to long-acting inhalers is associated with an increased incidence of acute exacerbation-related hospitalization or ED visits for treatment-naïve patients with COPD. Efforts to improve medication adherence even at the beginning of treatment may help reduce COPD exacerbations.

## Figures and Tables

**Table 1:** Clinical characteristics by medication adherence for patients with COPD naïve to long-acting inhalers.

Characteristics	All Patients (*n* = 3959)	MPR ≥ 80% (*n* = 2390)	MPR < 80% (*n* = 1569)	*p*-Value
Sex				0.0025
Women	925 (23.4)	519 (21.7)	406 (25.9)	
Men	3034 (76.6)	1871 (78.3)	1163 (74.1)	
Age, years				0.0003
40–49	111 (2.8)	78 (3.3)	33 (2.1)	
50–59	457 (11.5)	284 (11.9)	173 (11.0)	
60–69	1093 (27.6)	706 (29.6)	387 (24.7)	
70–79	1598 (40.4)	918 (38.4)	680 (43.3)	
80 or more	700 (17.7)	404 (16.9)	296 (18.9)	
Type of coverage				0.5307
Health insurance	3449 (87.1)	2093 (87.6)	1356 (86.4)	
Medical care	502 (12.7)	292 (12.2)	210 (13.4)	
Veteran welfare	8 (0.2)	5 (0.2)	3 (0.2)	
Number of outpatient clinics used				0.9219
1	3007 (76.0)	1814 (75.9)	1193 (76.0)	
2	952 (24.0)	576 (24.1)	376 (24.0)	
Number of comorbid diseases				0.4360
0	2171 (54.8)	1333 (55.8)	838 (53.4)	
1	1196 (30.2)	706 (29.5)	490 (31.2)	
2	476 (12.0)	283 (11.8)	193 (12.3)	
3	104 (2.6)	63 (2.6)	41 (2.6)	
4	12 (0.3)	5 (0.2)	7 (0.5)	
Number of inhalers used				<0.0001
1	3239 (81.8)	1909 (79.9)	1330 (84.8)	
2 or more	720 (18.2)	481 (20.1)	239 (15.2)	
Acute exacerbation				<0.0001
Incident	312 (7.9)	156 (6.5)	156 (9.9)	
Non-incident	3647 (92.1)	2234 (93.5)	1413 (90.1)	

Abbreviations: COPD, chronic obstructive pulmonary disease; MPR, medication possession ratio.

**Table 2:** Clinical characteristics and long-acting inhaler adherence according to the presence or absence of COPD exacerbation.

Characteristics	Acute Exacerbation of COPD		*p*-Value
	Presence (*n* = 312)	Absence (*n* = 3647)	
Sex			0.0084
Women	54 (17.3)	871 (23.9)	
Men	258 (82.7)	2776 (76.1)	
Age, years			<0.0001
40–49	1 (0.3)	110 (3.0)	
50–59	25 (8.0)	432 (11.9)	
60–69	61 (19.6)	1032 (28.3)	
70–79	144 (46.2)	1454 (39.9)	
80 or more	81 (26.0)	619 (17.0)	
Type of coverage			0.4520
Health insurance	267 (85.6)	3182 (87.3)	
Medical care	45 (14.4)	457 (12.5)	
Veteran welfare	0 (0.0)	8 (0.2)	
Number of outpatient clinics used			<0.0001
1	194 (62.2)	2813 (77.1)	
2 or more	118 (37.8)	834 (22.9)	
Number of comorbid diseases			<0.0001
0	130 (41.7)	2041 (56.0)	
1	95 (30.5)	1101 (30.2)	
2	60 (19.2)	416 (11.4)	
3	24 (7.7)	80 (2.2)	
4	3 (1.0)	9 (0.3)	
Number of inhalers used			<0.0001
1	207 (66.4)	3032 (83.1)	
2 or more	105 (33.7)	615 (16.9)	
Medication adherence			<0.0001
MPR ≥ 80%	156 (50.0)	2234 (61.3)	
MPR < 80%	156 (50.0)	1413 (38.7)	

Abbreviations: COPD, chronic obstructive pulmonary disease; MPR, medication possession ratio.

**Table 3:** Risk factors contributing to acute exacerbation in patients with COPD during follow-up.

Variables	Number (%)	Univariable Analysis		*p*-Value
		Odds Ratio	95% CI	
Medication adherence				<0.0001
MPR ≥ 80%	2390 (60.4)	1		
MPR < 80%	1569 (39.6)	1.58	1.25–1.99	
Sex				0.009
Women	925 (23.4)	1		
Men	3034 (76.6)	1.50	1.11–2.03	
Age, years				<0.0001
40–49	111 (2.8)	1		
50–59	457 (11.5)	6.37	0.85–47.50	
60–69	1093 (27.6)	6.50	0.89–47.36	
70–79	1598 (40.4)	10.89	1.51–78.61	
80 or more	700 (17.7)	14.39	1.98–104.51	
Number of comorbid diseases				<0.0001
0	2171 (54.8)	1		
1	1196 (30.2)	1.35	1.03–1.78	
2	476 (12.0)	2.26	1.64–3.13	
3	104 (2.6)	4.71	2.89–7.68	
4	12 (0.3)	5.23	1.40–19.56	
Number of inhalers used				<0.0001
1	3239 (81.8)	1		
2 or more	720 (18.2)	2.50	1.95–3.21	

Abbreviations: COPD, chronic obstructive pulmonary disease; CI, confidence interval; MPR, medication possession ratio.

**Table 4:** Relative risk of acute exacerbation per long-acting inhaler medication adherence in patients with COPD.

	Model 1		Model 2		Model 3	
	OR	95% CI	OR	95% CI	OR	95% CI
Medication Adherence						
MPR ≥ 80%	1		1		1	
MPR < 80%	1.55	1.22–1.95	1.57	1.24–1.99	1.68	1.33–2.14

Model 1, sex and age adjusted; Model 2, sex, age, and number of comorbid diseases adjusted; Model 3, sex, age, number of comorbid diseases, and number of inhalers used adjusted. Abbreviations: COPD, chronic obstructive pulmonary disease; CI, confidence interval; MPR, medication possession ratio.

## Data Availability

The data analyzed in this research are derived from the Healthcare Bigdata Hub (opendata.hira.or.kr (accessed on 1 June 2018)) in the Korean Health Insurance Review and Assessment Service database (www.hira.or.kr (accessed on 1 June 2018)). Authors had access to the study data for the purposes of this work only. Therefore, the data cannot be broadly disclosed or made publicly available at this time. Access to each database can be requested via the respective websites.
